# Troubleshooting during a challenging high-risk pacemaker lead extraction: a case report and review of the literature

**DOI:** 10.1186/s13104-015-1034-y

**Published:** 2015-03-25

**Authors:** Jacques Rizkallah, William Kent, Vikas Kuriachan, John Burgess, Derek Exner

**Affiliations:** Libin Cardiovascular Institute of Alberta, University of Calgary, TRW GE62, 3280 Hospital Drive NW, Calgary, T2N 4Z6 AB Canada

**Keywords:** Lead extraction, Hybrid surgery, Laser sheath, Sternotomy, Device infection

## Abstract

**Background:**

The use of cardiac implantable electrical devices continues to increase with the validation of new beneficial indications. While the risks of device implantation decreased significantly over time, significant risk remains associated with their extraction when indicated. A high-risk pacemaker lead extraction case is described, wherein a chronically implanted lead that had perforated the right atrium was successfully removed without the need for cardiopulmonary bypass. In this report we share our approach to this challenging extraction case and describe an infrequently utilized off-pump hybrid technique that we term the “lead-inverting stitch”.

**Case presentation:**

A 74 year-old Caucasian woman with complete heart block and remote pacemaker implantation presents with a swollen and erythematous infected pacemaker pocket necessitating device extraction. Chest computerized tomographic imaging revealed a chronically perforating right atrial lead tip approximately 2 cm within the pericardial space. A successful hybrid transvenous and open surgical extraction approach was undertaken without the need for cardiopulmonary bypass; this was made possible due to a successfully positioned “lead-inverting stitch”.

**Conclusion:**

Implantable cardiac electrical device infections are amongst the most dreaded post implant complications. Risks of device extraction are further complicated in cases of chronic lead perforations. Extraction strategies that avoid cardiopulmonary bypass initiation are preferred.

## Background

The use of cardiac implantable electrical devices (CIEDs) continues to increase with the validation of new beneficial indications. While the risks of device implantation decreased significantly over time, significant risk remains associated with their extraction when indicated. A high-risk pacemaker lead extraction case is described, wherein a chronically implanted lead that had perforated the right atrium was successfully removed without the need for cardiopulmonary bypass (CPB). In this report we share our approach to this challenging extraction case and describe an infrequently utilized off-pump hybrid technique that we term the “lead-inverting stitch”.

## Case presentation

**Figure 1:**
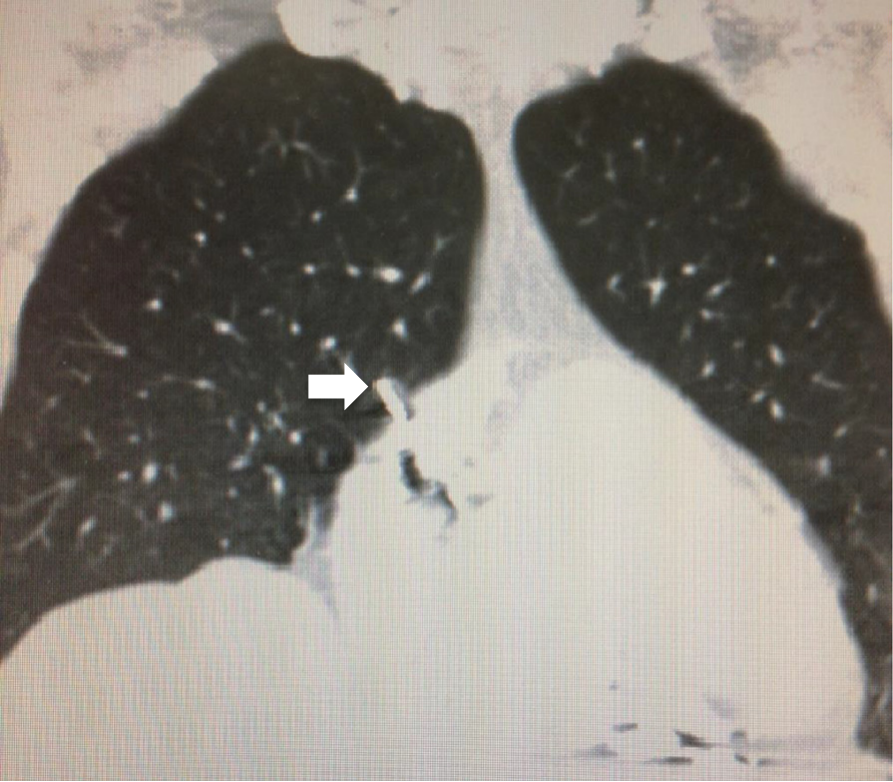
**Non-contrast computerized tomography image depicting the extravascular portion of the right atrial pacemaker lead (arrow).**

A 74 year-old Caucasian woman with complete heart block had dual chamber pacemaker implanted in 1990. Her initial system included a Medtronic 4504 passive fixation atrial lead and a Medtronic 4004 passive fixation ventricular lead. She subsequently underwent replacement of her pulse generator in 1998 and insertion of a new Medtronic 5568 active fixation atrial lead and a Medtronic 4068 ventricular leads in August 2003 due to lead failure. She underwent a subsequent generator change in June 2011. The original atrial and ventricular leads were abandoned when the new leads were added in 2003. All procedures had been undertaken at a referring institution and she had not had any prior open-heart surgical procedures. The patient presented at her home hospital with a swollen and erythematous pacemaker pocket in February 2014. Blood cultures did not grow any bacterial or fungal organisms. A trans-esophageal echocardiogram was performed to assess for vegetations on the leads. No vegetations were identified, but there was suspicion that one her atrial lead was extravascular. She underwent chest computerized tomographic imaging and this study revealed that one of her right atrial lead tips was approximately 2 cm within the pericardial space (Figure 1). The patient was treated with vancomycin and ceftriaxone based on a diagnosis of suspected pacemaker pocket infection. She underwent pacemaker pocket exploration and debridement, but extraction was not undertaken due to the increased risks involved with the lead being extra-vascular. No organism was identified from samples of fluid cultured during that initial debridement procedure. Despite that debridement procedure and prolonged antibiotics there was clinical recurrence of the infection and the patient was subsequently transferred to our center for complex lead extraction.

**Figure 2:**
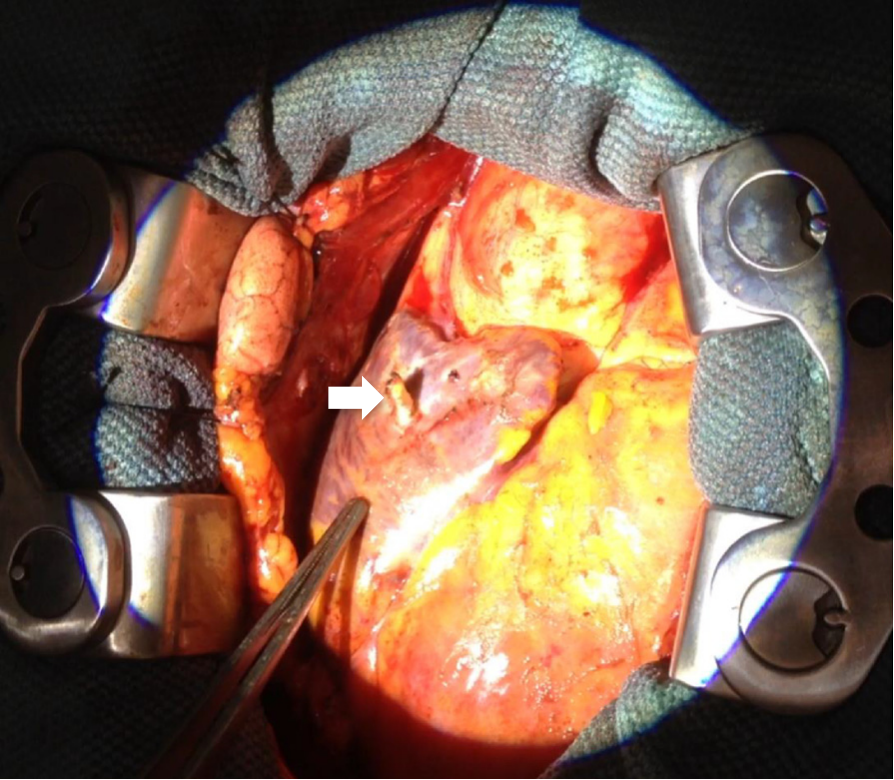
**Midline sternotomy depicting the pacemaker lead perforation through the right atrium (white arrow).**

**Figure 3:**
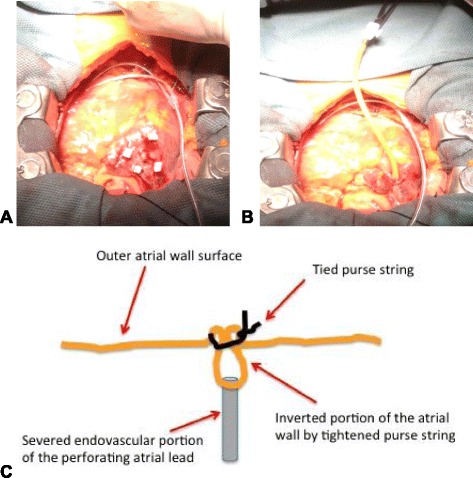
**Illustration of the “lead inverting stitch”. A.** Purse-string suture applied around the perforating lead. **B.** Atrial lead-tip severed with heavy scissors followed by inversion of the remnant atrial lead fragment and surrounding scar beneath the tightened purse-string. Note: the epicardial pacing lead is already in place. **C.** Cross-section illustration of the severed and inverted atrial lead following tightening of the purse string around the lead (i.e., “lead inverting stitch”).

To minimize the risk of vascular disruption and tamponade given the chronic right atrial lead perforation, a combined transvenous and open surgical extraction approach was undertaken. Using a dedicated hybrid operating theatre with cardiopulmonary bypass support if necessary, midline sternotomy was performed. Dense adhesions were found within the pericardial space consistent with likely prior pericarditis. Given the location of the RA lead, care was taken to leave the right atrial dissection until all other vascular structures were exposed. With uneventful dissection of the right atrium, direct visualization of the protruding passive fixation lead was obtained (Figure 2). To avoid the risks associated with cardiopulmonary bypass and given the good visualization of the perforating atria lead, an off-pump technique was considered using a “lead-inverting stitch”. To achieve hemostatic control over the region of the atrial wall where the lead exited, a purse-string stitch was placed circumferentially around the lead tip using 3–0 prolene with multiple pledgets (Figure 3A). The externalized tip of the perforating atrial lead was then mobilized by cutting a rim of surrounding atrial tissue. This allowed the end of the lead to be dunked within the right atrial chamber while the purse-string suture was tightened for hemostasis (Figure 3B and C). With this “lead inverting stitch”, the lead was free within the right atrium and could now be safely extracted using transvenous laser extraction techniques.

With the heart partially verticalized, the lateral wall of the left ventricle was exposed and an epicardial lead (Medtronic 4968) was placed. It was then tunnelled through the subcutaneous tissue above the rectus sheath, secured and connected to the new pacemaker. The new pacemaker was then placed in a newly created pocket, below the left costal margin. The sternotomy wound was covered with sterile surgical towels.

The infected pacemaker pocket was then opened and direct visualization confirmed the presence of a small amount of murky fluid. This fluid was sent for culture. The pocket was then extensively debrided and the four transvenous pacemaker leads were prepared for complex extraction in a standard manner beginning with retraction of the distal screw to facilitate separation of the active fixation lead tip from the myocardium interface during laser extraction. A purse string suture was subsequently positioned along the pectoral muscle around each lead at its insertion site to avoid any significant bleeding when the leads are completely removed. The lumen of each lead was then probed for patency with a standard stylet to ensure that a lead-locking-stylet can be utilized and advanced deep into the lead lumen for adequate gripping during the extraction process. Appropriate sizing of each lumen and distal delivery of the locking stylets was subsequently performed. A long silk suture thread was then tied to the outside of the lead near its insertion site, extended along the length of the lead, and tied to its end to provide further support along with the lead-locking-stylet when applying traction on the lead during laser extraction. Now that the leads have been prepared, transvenous lead extraction with a #14 French laser sheath was attempted. Due to the presence of multiple biding sites, up-sizing to a #16 laser sheath was required along with the use of a less compliant outer sheath (Visi-sheath). All the leads were removed entirely, with the perforating atrial lead removed last. There was no hemodynamic compromise throughout the procedure and no mechanical complications related to the procedure. The “lead inverting stitch” retained its integrity and hemostasis was preserved throughout the transvenous lead extraction.

Following successful lead extraction, the median sternotomy and old pacemaker pocket incisions were closed in a standard fashion and the patient was transferred to the intensive care unit for post-operative monitoring. The total procedure time from incision to wound closure was 5 hours and 24 minutes. Post-operative course was complicated with hypoxia secondary to atelectasis and aspiration pneumonia that resolved after 5 days. The patient was discharged back to her home hospital for ongoing rehabilitation.

## Discussion

A high-risk pacemaker lead extraction case is described, wherein a chronically implanted (>20 years) passive fixation lead that had perforated the right atrium was successfully removed without the need for CPB. To our knowledge such combined surgical and transvenous (hybrid) lead extraction procedures are typically completed with the patient on cardiopulmonary bypass. Our case presentation highlights how an off-pump hybrid approach utilizing a “lead-inverting stitch” can be considered and is likely preferred since it allows for an extravascular lead to be safely removed while avoiding the risks associated with cardiopulmonary bypass. This less invasive approach should be considered if adequate visualization of the perforating lead can be achieved.

The use of CIEDs continues to increase yearly with the validation of new beneficial and life saving clinical indications. In 2009 alone over 1.3 million CIEDs were implanted world-wide [1]. While the risks of device implantation have decreased significantly over time, there are significant risks associated with the removal of implanted devices as illustrated in the described case.

Based on a recent Danish nationwide cohort of over 5,000 consecutive patients undergoing CIED placement nearly 10% of recipients experienced at least one complication. Risk factors for complications included patient factors, an implant centre annual volume of < 750 CIED procedures, an annual operator volume <50 CIED procedures, system upgrade or lead revision and emergency or out-of-hours procedures. The most common major complication was lead related re-intervention, followed by infection. Cardiac perforation was identified in 0.6% of cases [2].

Complete CIED system removal, typically related to infection, is problematic. In high-volume centers, the risk of lead extraction still exceeds 5% and includes both major (1.8%) and minor (3.6%) complications [3]. In more typical centers the risk are likely much higher. For example, the risk of complications with lead revisions has been shown to be as high as 14.5%, with 7.25% of these being categorized as major complications [4].

Predictors of major complications with transvenous lead extraction include a history of cerebrovascular disease, an ejection fraction <15%, a reduced platelet count, an international normalized ratio >1.2, the need for mechanical sheaths or powered sheaths for lead removal [3,4]. Predictors of all-cause mortality within 30 days of extraction include a low weight, end-stage renal disease, higher New York Heart Association function class, lower haemoglobin, lead extraction for infection and extraction of a dual-coil implantable cardioverter defibrillator leads [3]. Thus, it is essential to carefully weigh the benefit and risk ratio when considering a patient for extraction.

Most leads are presently removed via transvenous extraction. In experienced centers procedural success exceeds 95.1% [3]. The presence of a lead or portion of it outside of the normal vascular space is an absolute contra-indication to transvenous lead extraction [5]. The case described is an example of such a patient. These patients are at much higher risk due to the potential for rapid exsanguination. A hybrid lead extraction approach involving midline or minimally invasive right thoracotomy in addition to transvenous extraction has been described and is reserved for high-risk cases where surgical intervention is deemed necessary for patient safety and to achieve clinical success [6,7].

Hybrid lead extraction procedures are not devoid of additional risks compared to isolated transvenous lead removal, particularly when cardiopulmonary bypass (CPB) is required. The incidence of intra- and post-operative complications associated with CPB, including mortality, increase exponentially with age [8]. The adage of “less is better” applies to every step of a hybrid lead extraction procedure. The described surgical technique of a “lead inverting stitch” allows the safe removal of an extravascular lead without the need for CPB; initiating patients on CPB is commonly done nowadays during hybrid extraction procedures where an atriotomy is necessary to surgically removal a perforating lead. This “lead inverting stitch” offers an alternative surgical approach in cases where avoidance of CPB is desirable. While the described technique is by no means infallible, this case illustrates that with adequate direct visualization, careful monitoring and direct surgical standby an extravascular lead can be removed without the initiation of CPB. Further, the described hybrid procedure was reasonably time efficient, with a procedural time of 5 hours and 24 minutes versus mean operative times of 13 hours when CPB is used as part of hybrid extraction procedures [6].

## Conclusion

Cardiac device infections represent serious complications, since complete system removal is typically necessary. Complex lead extraction is further complicated in cases, like that presented, where the lead or part of the lead is extra-cardiac. Recognition of this situation pre-operatively is essential to appropriate operative planning. A hybrid approach that avoids the need for cardiopulmonary bypass, like that described in this case, is preferred in order to minimize risk.

## Consent

Written informed consent was obtained from the patient for publication of this case report and any accompanying images. A copy of the written consent is available for review by the Editor-in-Chief of this journal.

## Abbreviations

CIEDs: Cardiac implantable electrical devices
CPB: Cardiopulmonary bypass
